# Consistency between headache diagnoses and ICHD-3 criteria across different levels of care

**DOI:** 10.1186/s10194-024-01937-6

**Published:** 2025-01-09

**Authors:** Lucas Hendrik Overeem, Marlene Ulrich, Mira Pauline Fitzek, Kristin Sophie Lange, Ja Bin Hong, Uwe Reuter, Bianca Raffaelli

**Affiliations:** 1 Department of Neurology, Charité – Universitätsmedizin Berlin, corporate member of Freie Universität Berlin and Humboldt-Universität zu Berlin, Berlin, Germany; 2 International Graduate Program Medical Neurosciences, Charité – Universitätsmedizin Berlin, corporate member of Freie Universität Berlin and Humboldt-Universität zu Berlin, Berlin, Germany; 3 Junior Clinician Scientist Program, Berlin Institute of Health at Charité (BIH), Berlin, Germany; 4 Clinician Scientist Program, Berlin Institute of Health at Charité (BIH), Berlin, Germany; 5 Universitätsmedizin Greifswald, Greifswald, Germany

**Keywords:** Diagnoses, Headache, ICHD-3, Levels of care, Lifting the burden

## Abstract

**Background:**

Diagnosing headache disorders poses significant challenges, particularly in primary and secondary levels of care (PSLC), potentially leading to misdiagnosis and underdiagnosis. This study evaluates diagnostic agreement for migraine, tension-type headache (TTH), and cluster headache (CH) between PSLC and tertiary care (TLC) and assesses adherence to the International Classification of Headache Disorders 3rd edition (ICHD-3) guidelines.

**Methods:**

A retrospective, cross-sectional analysis was conducted at Charité - Universitätsmedizin Berlin’s tertiary headache center. The patients’ self-reported diagnoses from the PSLC were compared with those in TLC and with ICHD-3 criteria. Cohen’s kappa (κ) and R² were used to assess diagnostic agreement.

**Results:**

Among 1,468 patients (43.4 ± 14.4 years; 74.5% women), 69.5% reported a diagnosis in PSLC, and 99.5% were diagnosed at their first TLC visit. Overall agreement between PSLC and TLC was 80% (κ = 0.55; R²=30%). Agreement between the PSLC and ICHD-3 was 77% for migraine, 82% for TTH, and 96% for CH (κ = 0.65; R²=41%). TLC diagnoses aligned with ICHD-3 in over 90%.

**Conclusion:**

Our findings indicate a significant degree of diagnostic agreement across different levels of care according to the ICHD-3 guidelines. However, there remains insufficient reliability in clinical diagnostics, highlighting the need for continued efforts to improve the early recognition and diagnostic accuracy and consistency of primary headaches to optimize patient care and treatment outcomes in Germany.

**Supplementary Information:**

The online version contains supplementary material available at 10.1186/s10194-024-01937-6.

## Background

Headache disorders are one of the most common medical conditions worldwide, with more than half of all adults having experienced a headache at least once within the past year [1]. Among the various types of primary headache disorders, tension-type headache (TTH) is the most prevalent one, followed by migraine. The global one-year prevalence of TTH is estimated to be around 30% [1], while migraine affects approximately 15% of the global population a year [1]. Less prevalent headache disorders include trigeminal autonomic cephalalgias (TAC), with cluster headache (CH) being the most common in this group and affecting roughly 0.1% of the global population [2].

Despite the high prevalence, burden, and significant socioeconomic impact, headache disorders are frequently underdiagnosed and undertreated [3–5]. The correct headache diagnosis can pose challenges due to the diverse range of underlying causes and the subjective nature of pain perception [6, 7]. Overlapping symptoms among different types of headache can complicate accurate diagnosis, leading to mismanagement or delayed treatment [6–8]. Furthermore, an accurate diagnosis requires a careful and often time-consuming interview to detail the characteristics of the headache. This process is particularly challenging due to the limited time resources available, especially in primary and secondary levels of care (PSLC) [9].

The International Classification of Headache Disorders 3rd edition (ICHD-3) [10], provides criteria and guidelines for healthcare professionals to diagnose and classify headache disorders accurately. However, the extent to which these criteria are considered and adhered to across different levels of headache care remains unclear.

In Germany, headache services are structured into a tiered system comprising three levels of care. Primary care is typically provided by general practitioners in outpatient settings, offering initial headache evaluation and management. The second level, secondary care, involves consultation with board-certified neurologists or anesthesiologists with expertise in managing neurological or pain conditions, including headaches. The third level of care, tertiary headache care, includes specialized headache centers that provide advanced and multidisciplinary care for especially difficult-to-diagnose or treat patients [11, 12]. Referrals to tertiary headache centers predominantly originate from secondary care providers. However, direct referrals from primary care are also possible. Patients who are likely to be referred to level 3 include those with refractory disabling headaches, cluster headache, medication overuse headache, high or low CSF-pressure headaches, trigeminal neuralgia, rare headache disorders, and those with severe physical or psychological comorbidities, as well as cases with diagnostic uncertainty, a risk of serious underlying conditions, or who may participate in specific research projects [13]. Discrepancies in headache diagnoses between these levels of care may arise due to various factors, including differences in expertise, resources, and diagnostic approaches.

The ‘Lifting the Burden’ campaign emphasized that common primary headache disorders, such as migraine, TTH, and CH should be accurately recognized and managed at the primary care level, as most cases can be effectively treated in this setting. However, despite these recommendations, the current diagnostic process remains suboptimal in terms of both accuracy and duration. Launched in March 2004, this campaign focuses on gathering evidence of the global headache burden, raising awareness among key stakeholders, and advocating for evidence-based, cost-effective interventions to improve headache management and reduce public health impact [14]. Its goal is to drive meaningful change in policy and healthcare practices [14]. As part of this campaign, German headache centers have participated in international collaborations such as the Eurolight Project [3, 15], among others. This has ensured a better understanding of which improvements can be made in headache care service.

In a 2022 German survey, 74% of migraine patients reported receiving a physician’s diagnosis; however, for 36% of them, the diagnostic process took more than two years [16]. Similarly, in 2012, Radtke and Neuhauser found that 63% of migraine patients were correctly diagnosed according to ICHD-II criteria [17]. Over the past decade, advances in headache management, including the introduction of ICHD-3 guidelines and initiatives like “The Global Campaign against Headache - Lifting The Burden (LTB)”, have aimed to improve diagnosis and care [10, 14]. Steiner et al. 2019 stated, “Accurate diagnosis is essential for optimal headache care“ [13]. The authors additionally provided an overview of roles at each level. Reassessing diagnostic accuracy is crucial for identifying persistent challenges. “In the ongoing effort to improve headache patient care through accurate diagnostics across all levels of care, we aimed to assess the diagnostic accuracy of migraine, TTH, and CH in patients referred to a German tertiary headache center according to the ICHD-3 criteria. Additionally, to emphasize the importance of consistent and accurate diagnosis for optimizing patient outcomes, we sought to identify potential pitfalls in applying diagnostic criteria and their impact on diagnostic accuracy.”

## Methods

### Study design & participants

This retrospective, cross-sectional study included patients who visited the tertiary headache center of Charité – Universitätsmedizin Berlin for the first time between December 2015 and January 2023.

In Germany, a referral from either primary or secondary care is a prerequisite for accessing tertiary care services. Prior to initial presentation, all new patients were asked to complete a headache history and symptom questionnaire at home, which serves as a preliminary step in their assessment process, obtaining e.g. data on prior diagnoses. While completing the questionnaire is entirely voluntary, we highly encourage it to facilitate the diagnostic process.

During the initial consultation at our headache center, the physician in charge collects a thorough medical history and performs a physical and neurological examination. This physician is either a board-certified neurologist with advanced training in headache medicine or a neurology resident under close supervision. Based on the findings of this comprehensive assessment, a diagnosis or provisional diagnosis is provided, irrespective of any diagnoses previously made at other levels of care. After this first consultation, the tertiary care physician communicates their findings, diagnosis, and recommendations to the referring physician via a standardized doctor’s letter.

From our patient registration log, we identified patients who received a first consultation at our headache center within the selected timeframe. We excluded patients if the questionnaire and/or doctor’s letter were unavailable. Additionally, patients who visited our center for non-headache disorders (e.g. facial pain or visual snow syndrome) were excluded for the purpose of this study.

### Outcomes

The primary outcomes of this study are the levels of agreement for the diagnoses of migraine, TTH, and CH between the PSLC and TLC as well as between PSLC and TLC and the ICHD-3 criteria.

The secondary outcomes include the identification of headache characteristics that might contribute to inconsistencies between diagnoses and compliance with ICHD-3 criteria. Due to the low incidence of disagreement between the TLC and the ICHD-3 criteria, this secondary endpoint was only obtained for the diagnoses from the PSLC.

The definitions of agreement and disagreement were as followed:

#### Positive Agreement

Agreement on the presence of migraine, tension-type headache (TTH), or cluster headache (CH).

#### Negative Agreement

Agreement on the absence of migraine, TTH, or CH.

#### Positive Disagreement

Disagreement about the presence of migraine, TTH, or CH according to a more specialized level of care or the gold standard (ICHD-3). This is also known as a missed diagnosis (missed diagnosis).

#### Negative Disagreement

Disagreement about the absence of migraine, TTH, or CH according to a more specialized level of care or the gold standard (ICHD-3). This is also known as a misdiagnosis (misdiagnosis).

In both the positive agreement and positive disagreement, diagnoses in the TLC had to fulfill all ICHD-3 criteria. For negative disagreement, diagnoses had to fulfill less than all but one of the ICHD-3 criteria.

### Data sources & Variables

We collected data from self-reported anamnesis questionnaires and doctor’s letters. To digitalize the paper questionnaires, we employed the semi-automated form processing application TeleForm 22.1 (Open Text Corp., Waterloo, Canada).

Variables obtained from the self-reported anamnesis questionnaires for this study included: birth year, sex, height, weight, disease duration, and the existence of any prior headache diagnosis and non-headache diagnosis. Variables obtained from the standardized doctor’s letters included: headache diagnoses (definite or suspected), headache frequency, headache onset, headache location, occurrence of migraine aura, pain quality, attack duration, and headache intensity measured using the numerical analog scale (NRS) on a scale from one to ten. In addition, we collected information about accompanying symptoms, including photophobia, osmophobia, phonophobia, nausea, and vomiting as well as autonomic symptoms, including conjunctival injection, lacrimation, nasal congestion, rhinorrhea, eyelid oedema, sweating, miosis, and ptosis.

The primary outcomes of this study are the diagnoses made across different levels of care. We defined the patients’ self-reported diagnoses from the PSLC as filled in on the questionnaire as the pre-existing PSLC diagnoses and the diagnoses reported in the doctor’s letter as TLC diagnoses. While stratification by primary and secondary care levels could provide more granular insights, it also introduces potential bias. Specifically, the data we have only reflect the referring physician at the time of the patient’s referral to our center and do not account for any prior visits to other levels of care. To minimize bias, we have combined primary and secondary care into a single category (PSCL) for the main analysis. However, stratified analyses by care level are provided in the supplementary material for additional context, though these should be interpreted with caution due to the aforementioned limitations. In this study, we primarily focus on the diagnoses of migraine (G43.-), TTH (G44.2), and CH (G44.0). Diagnoses falling under G44.1, G44.3 to G44.8, and R51 are defined as “others”.

Based on the variables collected from the standardized doctor’s letters, we assessed which patients met the ICHD-3 criteria for migraine, TTH, and/or CH or probable migraine, TTH, and/or CH. In the case of probable diagnoses, no alternative diagnosis fulfilling all criteria was permitted.

We double-checked cases with a positive or negative disagreement between the headache specialist and the ICHD-3 diagnosis. If the headache could be better explained by another diagnosis, the ICHD-3 diagnosis was corrected. For patients who explicitly described two or more types of headache, all types of headaches were assessed separately.

Missing data on self-reported diagnoses from the PSLC were interpreted as the absence of such diagnoses. Similarly, missing information on symptoms described in doctors’ letters was assumed to indicate the absence of those symptoms. To ensure data quality, patients with more than 10% missing information were excluded from the analysis.

### Statistical methods

The sample size of this study was determined by the availability of data rather than through a priori calculation. The data collection process was constrained by the number of eligible participants who met the inclusion criteria during the considered study period. We report our categorical variables in count (%) and continuous variables in mean ± standard deviation (SD). For statistical analysis, we used SPSS 29.0.0.0 (241) (IBM Corp., Armonk, NY, USA).

Based on the actual sample size, we assumed the continuous variables to be normally distributed according to the central limit theorem [18]. To compare groups, we used a Chi-Square Test, an Independent Samples T-Test, or an ANOVA as appropriate. Cohen’s kappa (κ) statistic measures the level of agreement between two raters. Since Cohen’s kappa is not directly interpretable, we also provided the coefficient of determination (R^2^) as an additional measure. R^2^ is calculated by squaring the κ value, indicating the proportion of variance explained by the agreement between raters. It ranges from 0 (0%, no agreement) to 1 (100%, perfect agreement). It is interpreted as follows:

- κ ≤ 0.20 indicates no agreement (R^2^ = 0–4% of the data are reliable),
- κ = 0.21–0.39 indicates minimal agreement (R^2^ = 4–15% reliability),
- κ = 0.40–0.59 indicates weak agreement (R^2^ = 15–35% reliability),
- κ = 0.60–0.79 indicates moderate agreement (R^2^ = 35–63% reliability),
- κ = 0.80–0.90 indicates strong agreement (R^2^ = 64–81% reliability),
- κ ≥ 0.90 indicates almost perfect agreement (R^2^ = 82–100% reliability) [19].

While there is no strict cut-off, McHugh suggested that a minimum R^2^ of 80% (κ ≥ 0.89) agreement can be considered acceptable [19]. By using both κ and R^2^, we provide a more comprehensive understanding of the level of agreement and the proportion of reliable data. Cohen’s kappa.

A multivariable logistic regression with backward stepwise elimination by Wald was used to identify which symptoms were associated with the likelihood of diagnostic disagreement between the diagnoses from the PSLC and the ICHD-3. To minimize bias from concurrent headaches, we included only patients who met the criteria for one or none of migraine, TTH, or CH. Here we report the odds ratio (OR) and 95% confidence interval (95% CI).

No corrections for potential confounding or effect modification were made. A p-value below 0.05 was considered statistically significant.

### Ethics

This study was approved by the Charité – Universitätsmedizin Berlin ethical committee (EA4/246/23). Due to the retrospective nature of this study, written informed consent from enrolled patients was not required under local regulations. This study is reported in accordance with the “Strengthening the Reporting of Observational Studies in Epidemiology” (STROBE) statement for cohort studies [20].

### Data Availability

The data that support the findings of this study are available from the corresponding author, upon reasonable request.

## Results

### Patient selection & characteristics

**Fig. 1:**
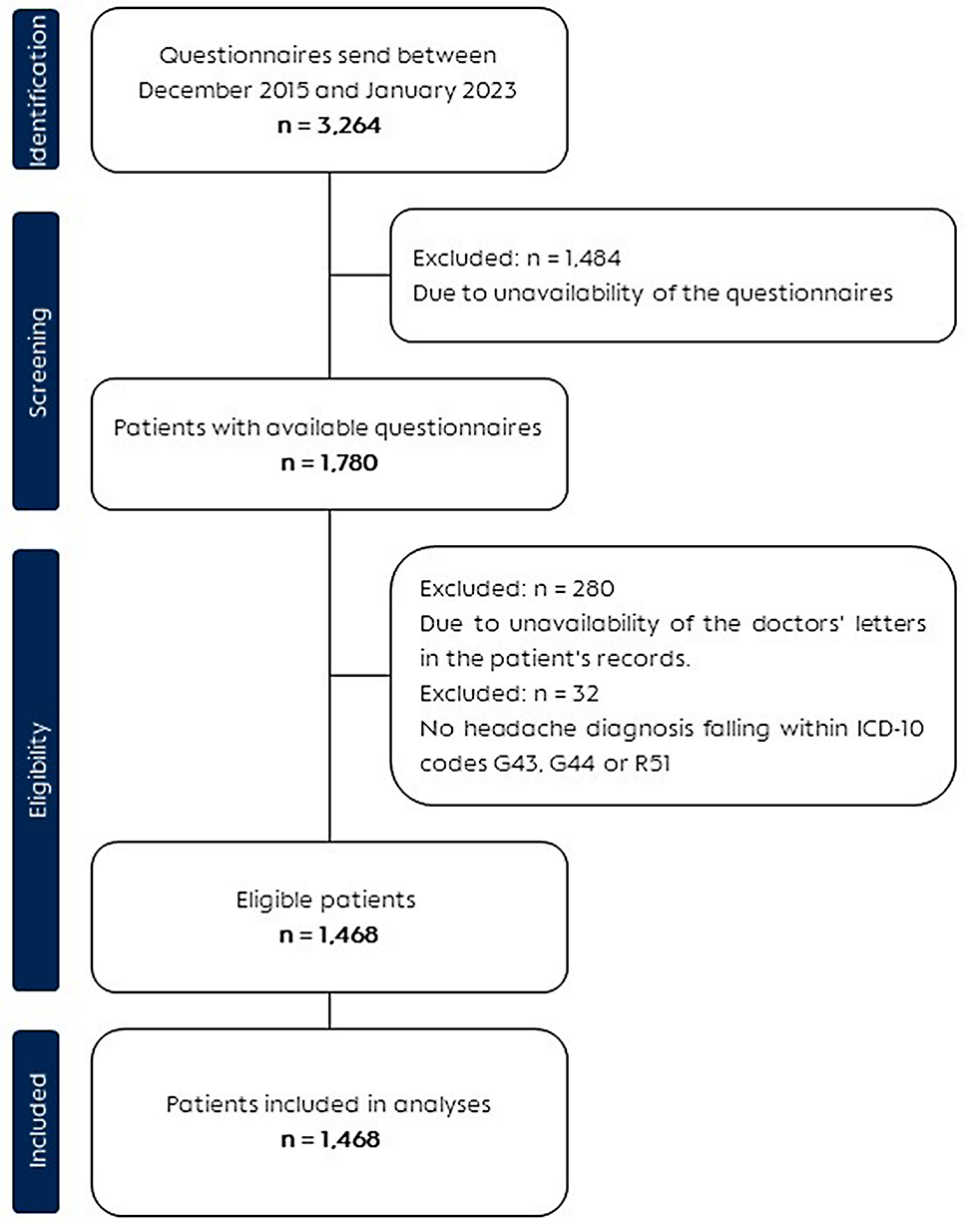
Flowchart of patient selection and inclusion. This flowchart details the stepwise selection of patients included in this study

From the 3,264 questionnaires that were sent to the patients between December 2015 and January 2023, 1,468 (44.0%) were included in this analysis, Fig. 1.

The cohort consisted of 1,094 (74.1%) women, the mean age was 43.4 ± 14.4 years. Further characteristics are reported in Table 1 and sTable 1.

**Table 1:** Patient characteristics

Characteristic	Total cohort *N* = 1,468
Age (years)	43.4 ± 14.4
Women	1,094 (74.5)
Age of headache onset (years)	24.7 ± 15.9
Disease duration (years)	18.6 ± 14.8
BMI Score (kg/m^2^)	25.1 ± 5.2
BMI Categories:	
18.5 Underweight	52 (3.5)
18.5–24.9Normal Weight	732 (49.9)
25.0–29.9Overweight	374 (25.5)
≥30Obese	198 (13.5)
Missing	112 (7.6)
Comorbid diseases:	
Anxiety	261 (17.8)
Depression	326 (22.2)
Diabetes	45 (3.1)
Heart disease	100 (6.8)
Hyperlipidemia	114 (7.8)
Hypertension	227 (15.5)
Sleep disorders	533 (36.3)
Stroke	33 (2.2)
Thyroid diseases	259 (17.6)

Values are given in count (%) or mean ± SD

### Headache diagnoses

#### Diagnoses from the primary/secondary level of care

A total of 1,020 patients (69.5%) reported to have received a headache diagnosis at the PSLC (Table 2). Patients referred by the secondary level of care were more likely to be diagnosed with migraine compared to patients referred from the primary level of care, X^2^(1, *N* = 1,468) = 5.2, *p* = 0.025, Table 2. Diagnoses stratified for the primary and secondary care can be found in sTable 2.

**Table 2:** Headache diagnosis from the PSLC and TLC

Diagnosis	PSLC	TLC
None	448 (30.5)	8 (0.5)
One diagnosis	725 (49.4)	1,206 (82.2)
Two diagnoses	268 (18.3)	244 (16.6)
Three diagnoses	27 (1.8)	10 (0.7)
Migraine	824 (56.1)	1,168 (79.6)
Tension-type headache	365 (24.9)	227 (15.5)
Cluster headache	111 (7.6)	62 (4.2)
Other headache	42 (2.9)	267 (18.2)

#### Diagnoses from the tertiary level of care

During the patients’ first presentation at our headache center, 1,460 (99.5%) patients were diagnosed with at least one headache diagnosis (Table 2). This number includes both definite and suspected diagnoses.

#### Classification of headache diagnoses based on the ICHD-3 criteria

Based on the reported headache characteristics and associated symptoms, 1,419 (96.7%) patients fulfilled all or all but one criteria for migraine, TTH, and/or CH (Table 3).

**Table 3:** Number of patients fulfilling the ICHD-3 diagnostic criteria for migraine, TTH, and cluster headache

ICHD-3 criteria	Fulfilling all*	Fulfilling all but one**
None	379 (25.8)	49 (3.3)
One diagnosis	1,066 (72.6)	1,166 (79.4)
Two diagnoses	23 (1.6)	247 (16.8)
Three diagnoses	-	6 (0.4)
Migraine	1,017 (69.3)	1,237 (84.3)
Tension-type headache	59 (4.0)	363 (24.7)
Cluster headache	36 (2.5)	78 (5.3)

* Fulfilling all criteria and were not better explained by another ICHD-3 diagnosis.

** Fulfilling all but one criteria indicates a probable diagnosis if not better explained by another ICHD-3 diagnosis

### Agreement between PSLC and TLC

Out of the 1,300 reported diagnoses for migraine, tension-type headache (TTH), and cluster headache (CH) in the PSLC, 948 (73%) were also confirmed in the TLC. The overall diagnostic agreement between the PSLC and TLC was 80%, classified as a “weak,” level of agreement with κ = 0.55 (0.52–0.57) and R²=30%. This indicates that the PSLC diagnoses only partially align with the TLC diagnoses, with the remaining 70% of the differences likely reflecting variations in diagnostic criteria interpretation, clinical judgment, or other systemic differences between the PSLC and TLC.

**Fig. 2:**
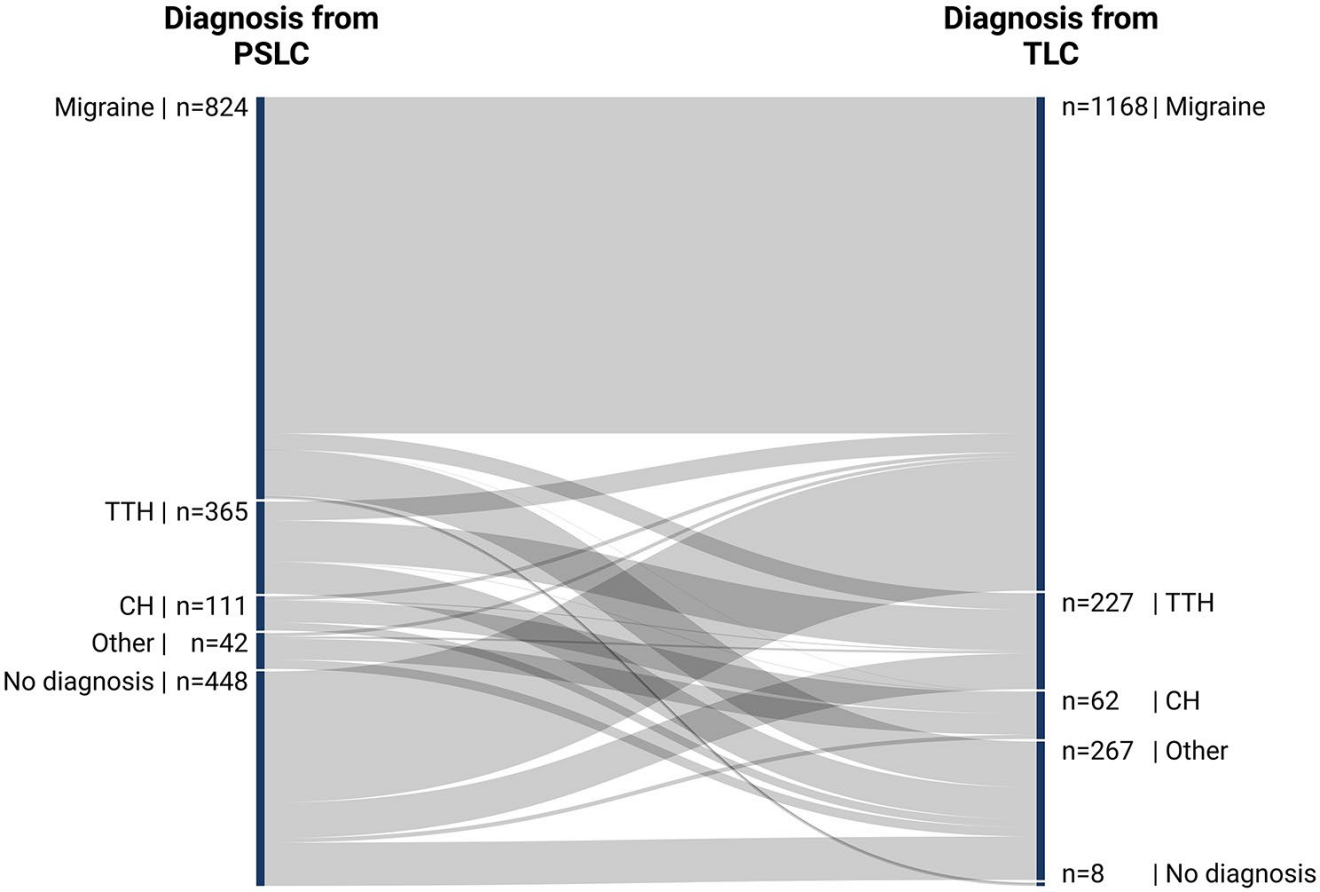
Sankey diagram visualizing the agreement of headache diagnoses among the PSLC and TLC. This Sankey diagram visualizes the diagnostic agreement of diagnoses between the primary and secondary level of care (PSLC) and tertiary level of care (TLC)

Figure 2 illustrates this consistency, while sTable 3 provides additional details.

#### Migraine

Of 824 migraine diagnoses in the PSLC, 800 (97%) were confirmed by the TLC, while 24 (3%) were not. Additionally, 368 patients (57%) not diagnosed with migraine in the PSLC were diagnosed with it in the TLC. This results in a “weak” agreement with κ = 0.42 (0.38–0.47) and R²=18%.

#### Tension-type headache

Among the 365 TTH diagnoses in the PSLC, only 97 (27%) were confirmed by the TLC, with 268 (73%) not corroborated. This suggests “no agreement,” as the κ for TTH was 0.17 (0.10–0.24) and R²=3%.

#### Cluster headache

Of 111 CH diagnoses in the PSLC, 51 (46%) were confirmed by the TLC, while 60 (54%) were not. Additionally, 11 patients (1%) not diagnosed with CH in the PSLC were later diagnosed with it in the TLC. This reflects a “weak” agreement with κ = 0.75 (0.47–0.67) and R²=32%.

**Fig. 3:**
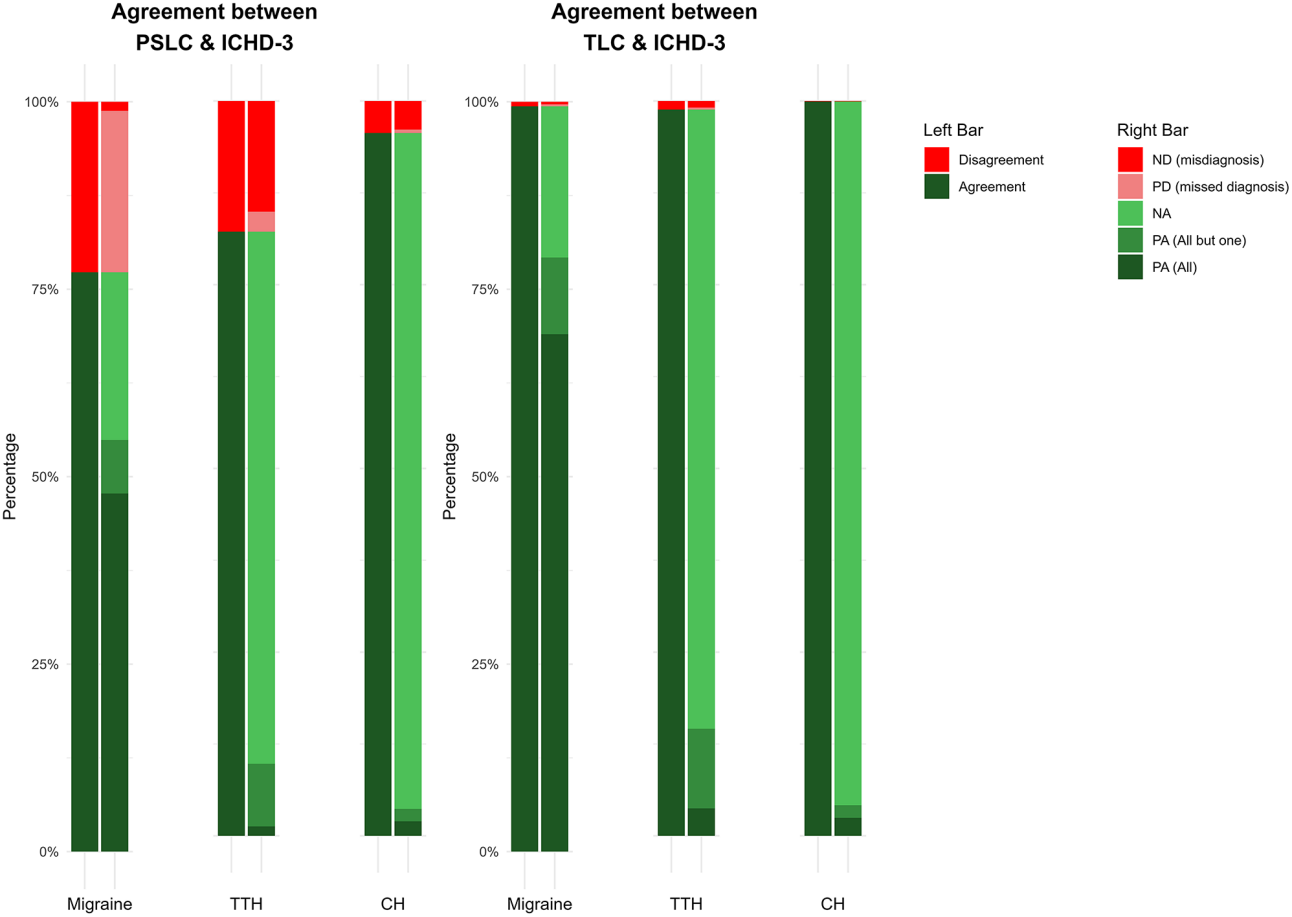
Bar chart of diagnostic agreement for headache at PSLC and TLC per ICHD-3 criteria. This grouped bar chart shows the accuracy of headache diagnoses made at the primary and secondary level of care (PSLC) and the tertiary level of care (TLC) against the third edition of The International Classification of Headache Disorders (ICHD-3) criteria for migraine, tension-type headaches (TTH), and cluster headaches (CH). PA = Positive Agreement; PD = Positive Disagreement; NA = Negative Agreement; ND = Negative Disagreement

The diagnoses provided in the PSLC and TLC were compared with those based on the ICHD-3 criteria. Figure 3 offers an overview of these agreements.

### Agreement between PSLC diagnoses and ICHD-3 guidelines

**Fig. 4:**
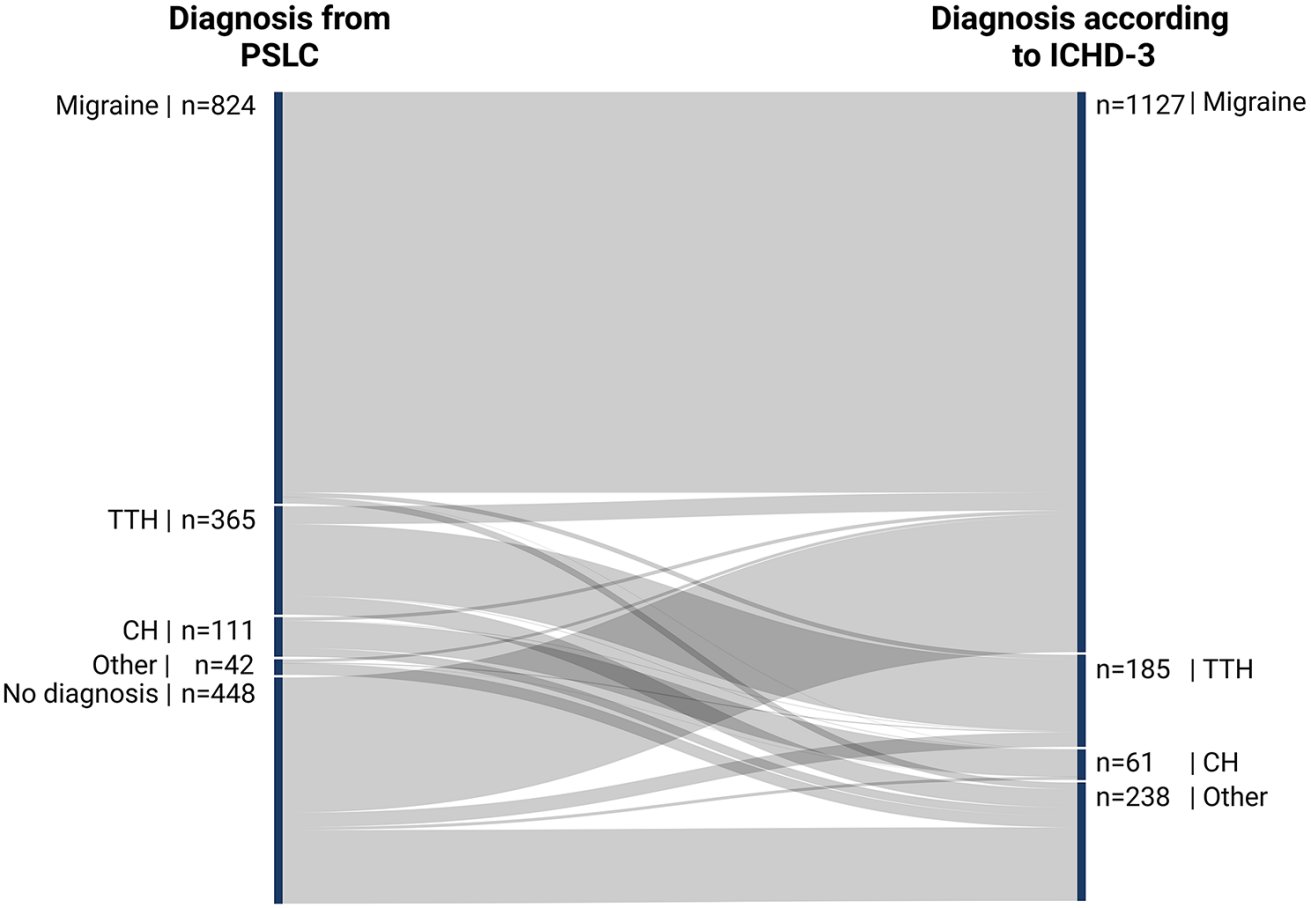
Sankey diagram visualizing the agreement of headache diagnoses among the PSLC and ICHD-3 diagnostic criteria. This Sankey diagram visualizes the diagnostic agreement of diagnoses given at the primary and secondary level of care (PSLC) accordingly to the third edition of The International Classification of Headache Disorders (ICHD-3)

The overall agreement between PSLC diagnoses of migraine, TTH, and CH with ICHD-3 criteria was 85%. Of the 1,300 diagnoses made in the PSLC, 1,004 (77%) met the ICHD-3 standards, reflecting a “moderate” agreement with κ = 0.65 (0.62–0.67) and R²=42%. This indicates that the TLC diagnoses align almost perfectly with the ICHD-3 criteria, with only 58% of the variability likely attributable to differences in the interpretation of diagnostic criteria, clinical judgment, or other minor factors. Figure 4 shows this concordance and sTable 4 provides further details.

#### Migraine

Among 824 patients diagnosed with migraine in the PSLC, 806 (98%) met ICHD-3 criteria, while 18 (2%) did not. An additional 316 patients (38%) who met all migraine criteria were not diagnosed accordingly. This results in a “weak” agreement with κ = 0.51 (0.47–0.56) and R²=26%.

#### Tension-type headache

Of the 365 patients diagnosed with TTH, 144 (40%) met ICHD-3 criteria, while 221 (60%) did not. Additionally, 40 patients (11%) meeting the criteria were not diagnosed with TTH. This results in a “weak” agreement with κ = 0.43 (0.37–0.49) and R²=18%.

#### Cluster headache

Out of 111 CH diagnoses, 54 (49%) met ICHD-3 criteria, while 57 (51%) did not. Furthermore, 7 patients (6%) meeting the criteria were not diagnosed with CH. This reflects a “moderate” agreement with κ = 0.61 (0.51–0.70) and R²=37%.

sTable 5a and sTable 5b provide the stratified diagnostic agreement for primary and secondary levels of care according to the ICHD-3 guidelines. There was no significant difference between the PLC (84.5% total agreement) and SLC (85.7% total agreement).

### Agreement between TLC diagnoses and ICHD-3 guidelines

**Fig. 5:**
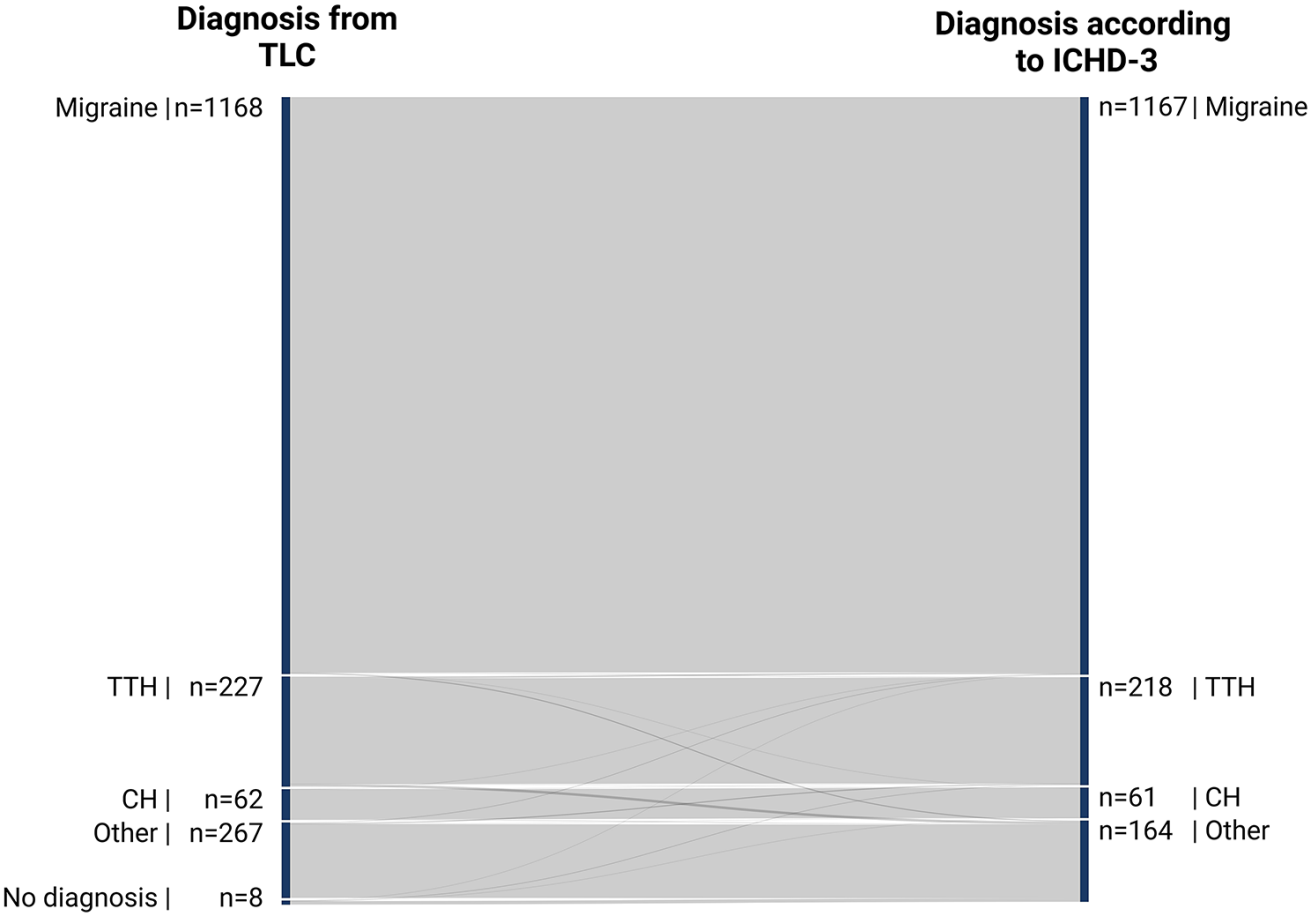
Sankey diagram visualizing the agreement of headache diagnoses among the TLC and ICHD-3 diagnostic criteria. this Sankey diagram visualizes the diagnostic agreement of diagnoses given at the tertiary level of care (TLC) accordingly to the third edition of the international classification of headache disorders

In the TLC, the overall agreement of migraine, TTH, and CH diagnoses with ICHD-3 criteria was 99%. Of 1,457 diagnoses, 1,438 (99%) were consistent with ICHD-3 guidelines, resulting in an “almost perfect” agreement with κ = 0.99 (0.98–0.99) and R²=97%. This indicates that the TLC diagnoses align with the ICHD-3 criteria, with only 3% of the differences can likely be explained by to variations in the interpretation of diagnostic criteria or clinical judgment. Figure 5 illustrates this alignment, and sTable 6 provides additional details.

#### Migraine

Among the 1,168 migraine diagnoses in the TLC, 1,163 (> 99%) met ICHD-3 criteria, while 5 (< 1%) did not. This reflects an “almost perfect” agreement with κ = 0.98 (0.97–0.99) and R²=96%.

#### Tension-type headache

Of 227 TTH diagnoses, 214 (94%) were consistent with ICHD-3 criteria, while 13 (6%) were not. This corresponds to κ = 0.96 (0.93–0.98) and R²=91%.

#### Cluster headache

Among 62 CH diagnoses, 61 (98%) met ICHD-3 criteria, yielding κ = 0.99 (0.98–0.99) and R²=98%.

### Associated factors for diagnostic disagreements

Given the underdiagnosis of migraine observed in our study, we examined which clinical symptoms may have contributed to this in the PSLC. In the multivariate analysis, we found that the absence of intense pain (VAS 4–9), photophobia, nausea, vomiting, or aura significantly increased the likelihood of missing a migraine diagnosis (sTable 7). Pain intensity (OR 2.27), vomiting (OR 1.54), and aura (OR 1.70) were identified as key factors.

TTH was found to be overdiagnosed in the PSLC. An analysis of contributing factors revealed that some patients were diagnosed with TTH even when the ICHD-3 criteria were not fully met. These factors included the absence of bilateral pain, pressing or tightening pain, moderate pain intensity (VAS 1–6), and no pain aggravated by physical activity (sTable 8). Among these, pain quality (OR 2.12) and pain intensity (OR 2.02) were the strongest contributors to overdiagnosis.

CH was also overdiagnosed in the PSLC. Our multivariate analysis identified that the presence of unilateral pain (OR 2.17) and ipsilateral autonomic symptoms (OR 2.04) significantly increased the likelihood of being misdiagnosed with CH in the PSLC (sTable 9).

## Discussion

Of the 1,468 patients visiting our tertiary headache center for the first time, 69.5% had previously received a headache diagnosis at the PSLC. At our center, 99.5% were diagnosed with at least one headache diagnosis during their initial visit. Migraine was underdiagnosed at the PSLC, while TTH was overdiagnosed. The coefficient of determination (R²) for migraine, TTH, and CH at the PSLC fell below the acceptable 80%, suggesting insufficient adherence to ICHD-3 guidelines in the PSLC setting. This highlights the need for improving diagnostic accuracy in primary and secondary headache care.

The global initiative against Headache, ‘Lifting The Burden,’ introduced the current framework of three levels of headache care [21]. The majority of people with headaches are diagnosed and treated at the primary and secondary level of care and only a fraction of those patients require tertiary headache care in a 90:9:1% split [22]. Therefore, it is essential that headaches are correctly recognized, diagnosed, and managed in the PSLC [23]. Accurate diagnosis ensures that the patient receives the appropriate treatment, such as pharmacological therapy or lifestyle modifications, which can significantly reduce the frequency and severity of headaches over time [24, 25]. Additionally, misdiagnosis or delayed diagnosis may lead to ineffective treatments, unnecessary investigations, and prolonged patient suffering [24, 25]. Therefore, diagnostic accuracy is essential for long-term management, outcomes, and prognosis.

In this study, 67% of patients presenting at a tertiary headache center received the same diagnosis they had previously been given at lower levels of care. While this indicates a substantial level of agreement, it also reveals that approximately one-third of these patients either received a new diagnosis or were diagnosed for the first time solely at the tertiary care level. This points to discrepancies or missed diagnoses in the primary and secondary care settings [26–28], emphasizing the need for improved diagnostic accuracy.

Migraine was the predominant diagnosis in our cohort at both the PSLC and TLC, but a notable discrepancy existed between the two levels. At PSLC, the likelihood of a migraine diagnosis when meeting ICHD-3 criteria was substantially lower compared to TLC, indicating underdiagnosis at PSLC. In addition, more than 60% of patients without a prior PSLC diagnosis met all ICHD-3 criteria for migraine, with about half reporting aura, which was not correctly identified or considered when making a diagnosis.

The issue of underdiagnosis of migraine is well-documented in previous studies. A large multi-center study across seven countries found correct migraine diagnoses by general practitioners in only 28% of cases, with similar trends observed at secondary care levels [4]. Studies from Russia, Italy, and China reported accurate prior diagnosis rates from 12 to 27% [29–31].

Comparing our findings with those previous reports, it seems that German physicians in the PSLC demonstrate above-average accuracy in diagnosing migraine. In line with this observation, a 2012 German study reported that 63% of individuals meeting the ICHD-2 criteria for migraine and consulting a physician were correctly diagnosed with migraine [17]. However, significant room for improvement remains, with minimal progress since 2012. Enhanced diagnostic strategies and awareness campaigns are needed, with some authors suggesting that most patients with disabling, episodic headaches should be diagnosed with migraine by default at the primary level [32].

Regarding specific migraine symptoms, a comprehensive review identified nausea, pain exacerbation by physical activity, and photophobia as the most sensitive clinical features for diagnosing migraine [33]. Rai et al. identified non-throbbing pain, non-temporal pain, and the first physician not being a neurologist as predictor factors for inappropriate migraine diagnosis [34]. Radtke and Neuhauser associated nausea/vomiting, photophobia/phonophobia, unilateral pain, and severe headache with recognition of migraine at PSLC in Germany [17]. Our findings align partially, indicating that moderate to severe pain, photophobia, nausea, vomiting, or aura increased correct diagnosis likelihood, while unilateral pain, throbbing pain, and phonophobia did not. While not all diagnostic features are mandatory for diagnosing migraine [10, 35], awareness of all associated symptoms may be beneficial for cases that do not meet al.l diagnostic criteria.

TTH appears to be often misdiagnosed in the PSLC. Among patients initially diagnosed with TTH in the PSLC, 13% should have been diagnosed with migraine instead and 69% with migraine alone according to the ICHD-3 criteria. Previous studies highlight this misdiagnosis tendency. The Spectrum study found 32% of TTH diagnoses were actually migraine [36]. In the TEDDI study, only 2.4% of patients diagnosed with TTH in the emergency department met all criteria and 7.6% met all but one criteria for TTH [37]. These findings underscore the importance of thorough evaluation and adherence to standardized diagnostic criteria to prevent misdiagnosis and ensure appropriate management of headache disorders, particularly in distinguishing between tension-type headache and migraine.

Regarding CH, studies in Italy, East Europe, and Spain reported high rates of misdiagnosis at initial consultations, with only a minority of CH cases correctly identified [38–40]. Interestingly, German physicians in PSLC seem to exhibit higher diagnostic accuracy, with a smaller proportion of CH cases misdiagnosed in our cohort. Nonetheless, there remains a need for improvement, as one out of five CH patients was still missed in PSLC.

The Aids to Management of Headache Disorders in Primary Care proposed that CH should be diagnosed in PSLC and managed in TLC [41]. Despite the recommendation that CH should “never be missed” in PSLC, it appears that the goal of a correct diagnosis at PSLC has not yet been fully achieved. Thus, there is still work to be done in terms of improving both physician education in PSLC and raising awareness among the general population [4, 39].

Though to a much lesser extent, misdiagnosis of CH has also been previously described, particularly among men with migraine [42]. In our cohort, ipsilateral autonomic symptoms were linked to CH misdiagnosis. While these symptoms are typical of CH, they can also appear in migraine and other headache disorders. For example, Karsan et al. reported cranial autonomic symptoms in 74% of migraine patients [43], and Togha et al. found a prevalence of 61% [44].

Another factor contributing to the under-recognition of primary headache disorders in PSLC could stem from the design of the International Classification of Headache Disorders (ICHD), which prioritizes specificity over sensitivity, potentially leading to under-diagnosis [24]. Consequently, the guidelines should be viewed as guidance rather than strict rules. It is important to acknowledge that some patients may not fully meet al.l criteria for a specific headache disorder, or criteria fulfillment may be unclear or vary between attacks [45].

Our findings may not be generalizable to all patients seen at PSLC, as this study focuses on those with potentially more complex diagnostic and treatment challenges. Furthermore, these results should not be viewed as a criticism of the diagnostic accuracy of PSLC physicians. Rather, the intent is to identify areas for potential improvement in diagnostic practices, with the aim of enhancing overall care quality and reducing unnecessary diagnostic delays.

The novelty of this study lies in its systematic evaluation of diagnostic practices for three major headache disorders across different levels of care in a large German population. In addition, we were able to identify key factors contributing to potential diagnostic errors. Our findings contribute to the broader scientific understanding by confirming and expanding upon results from similar studies conducted in other countries or with different designs. These insights have the potential to enhance the accuracy of headache diagnoses, improve treatment outcomes, optimize healthcare resource allocation, and promote a more standardized approach to headache care across all levels of the healthcare system.

### Recommendations for improving diagnostic consistency and accuracy

To enhance diagnostic consistency and accuracy in primary and secondary care settings, ongoing training and education are essential and have been shown to be effective [11]. This could involve expanding headache education during medical training, offering e-learning modules, and providing regular headache training programs for physicians of the PSLC [25]. The ICHD-3 diagnostic criteria can be challenging for non-neurologists, and the large number of distinct diagnoses may be overwhelming for daily use in PSLC settings. Therefore, developing a user-friendly format for clinical use might be beneficial and could help physicians to be more confident in making accurate diagnoses and reduce unnecessary referrals [33]. Diagnostic tools such as validated and standardized questionnaires or AI-assisted diagnostic support might be supportive in this. Additionally, it remains crucial to monitor diagnostic consistency across care levels and share the results with providers as part of a continuous improvement strategy. Research should also focus on identifying areas of disagreement in diagnoses between care levels to address these issues systematically [5].

### Strengths and limitations

A key strength of this study is its large sample size, allowing robust conclusions even for less common headache disorders. We thoroughly cross-referenced cases with discrepancies between TLC diagnoses and ICHD-3 criteria to mitigate potential errors.

However, some limitations exist. Reliance on patient-reported diagnoses from PSLC may introduce bias and under-documentation, potentially overestimating our results. The lack of detailed data regarding the reasons for patient referrals to our tertiary center may have introduced selection bias by disproportionately including cases with greater diagnostic complexity or uncertainty. However, identifying why those patients failed to get diagnosed in the PSLC was also part of this study’s aim. Doctor’s letters may also introduce reporting bias, as not all symptoms or their absence are consistently documented, despite a standardized template. This makes it challenging to determine whether symptoms were omitted during assessment or genuinely absent. A similar concern applies to the possibility of prior visits to another TLC. However, given that the nearest alternative center is *≈* 200 km away, such instances are very rare and unlikely to have a significant impact on our results. Additionally, the occurrence of multiple headache types described by patients could bias our prediction models. We minimized this potential bias by including only patients meeting none or only one of the ICHD-3 diagnoses of migraine, TTH, and CH.

## Conclusion

While there is notable consensus in diagnosis based on ICHD-3 guidelines, challenges persist, particularly in primary and secondary care, with migraine being underdiagnosed, tension-type headache misdiagnosed, and cluster headache exhibiting both misdiagnosis and under-recognition. These discrepancies underscore the need for comprehensive strategies encompassing improved physician education and heightened public awareness. Addressing these issues is crucial to enhancing diagnostic accuracy and ensuring optimal management of headache disorders.

## Electronic Supplementary Material

Below is the link to the electronic supplementary material.

Supplementary Material 1

## Data Availability

No datasets were generated or analysed during the current study.

## Abbreviations

ANOVA: Analysis of Variance
CH: Cluster Headache
CSF: Cerebrospinal Fluid
ICHD: 3–International Classification of Headache Disorders, 3rd edition
NRS: Numerical Rating Scale
PSLC: Primary and Secondary Levels of Care
R²: Coefficient of Determination
SPSS: Statistical Package for the Social Sciences
STROBE: Strengthening the Reporting of Observational Studies in Epidemiology
TAC: Trigeminal autonomic cephalalgias
TLC: Tertiary Level of Care
TTH: Tension–Type Headache
VAS: Visual Analog Scale
κ: Cohen’s Kappa (measure of agreement)
