# Supplementary material for: Consistency between headache diagnoses and ICHD-3 criteria across different levels of care
Source: J Headache Pain. 2025 Jan 9;26(1):6. doi: 10.1186/s10194-024-01937-6 (PMC11715415; doi:10.1186/s10194-024-01937-6)
Supplement: Supplementary file 1 — Supplementary Material 1 [file 10194_2024_1937_MOESM1_ESM.docx]

# Consistency between headache diagnoses and ICHD-3 criteria across different levels of care

# Supplementary material

Lucas Hendrik Overeem ^1,2^, Marlene Ulrich ^1^, Mira Pauline Fitzek ^1,3^, Kristin Sophie Lange ^1,4^, Ja Bin Hong ^1^, Uwe Reuter ^1,5^, Bianca Raffaelli ^1,4*^

**Author affiliations:**

1 Charité – Universitätsmedizin Berlin, Department of Neurology, 10117 Berlin, Germany

2 Doctoral Program, International Graduate Program Medical Neurosciences, Humboldt Graduate School, 10117 Berlin, Germany.

3 Junior Clinician Scientist Program, Berlin Institute of Health (BIH), 10117 Berlin, Germany.

4 Clinician Scientist Program, Berlin Institute of Health (BIH), 10117 Berlin, Germany.

5 Universitätsmedizin Greifswald, Greifswald, Germany.

**Correspondence to:**

*Bianca Raffaelli

Charité – Universitätsmedizin Berlin

Department of Neurology with Experimental Neurology

Charitéplatz 1

10117 Berlin, Germany

bianca.raffaelli@charite.de

# Content:

| sFigure 1: Diagnostic fulfillment per diagnostic criteria for probable migraine (A), probable TTH (B), and probable CH (C) | 3 |
| --- | --- |
| sTable 1: Headache characteristics | 5 |
| sTable 2. Pre-existing headache diagnosis from the primary and secondary level of care | 6 |
| sTable 3: Agreement of headache diagnosis from primary/secondary level of care relative to the tertiary level of care | 7 |
| sTable 4: Agreement of headache diagnosis from primary/secondary level of care relative to the ICHD-3 Guidelines | 8 |
| sTable 5a: Agreement of headache diagnosis from primary level of care relative to the ICHD-3 Guidelines | 9 |
| sTable 5b: Agreement of headache diagnosis from secondary level of care relative to the ICHD-3 Guidelines | 10 |
| sTable 6: Agreement of diagnosis from headache specialist according to the ICHD-3 Guidelines | 11 |
| sTable 7: Predictors for a positive disagreement in diagnosing migraine in the PSLC according to the ICHD-3 criteria, n=1042 | 12 |
| sTable 8: Predictors for a negative diagnostic disagreement of TTH in the PSLC according to the ICHD-3 criteria, n=1544 | 13 |
| sTable 9: Predictors for a negative diagnostic disagreement of CH in the PSLC according to the ICHD-3 criteria, n=1600 | 14 |

# sFigure 1: Diagnostic fulfillment per diagnostic criteria for probable migraine (A), probable TTH (B), and probable CH (C)


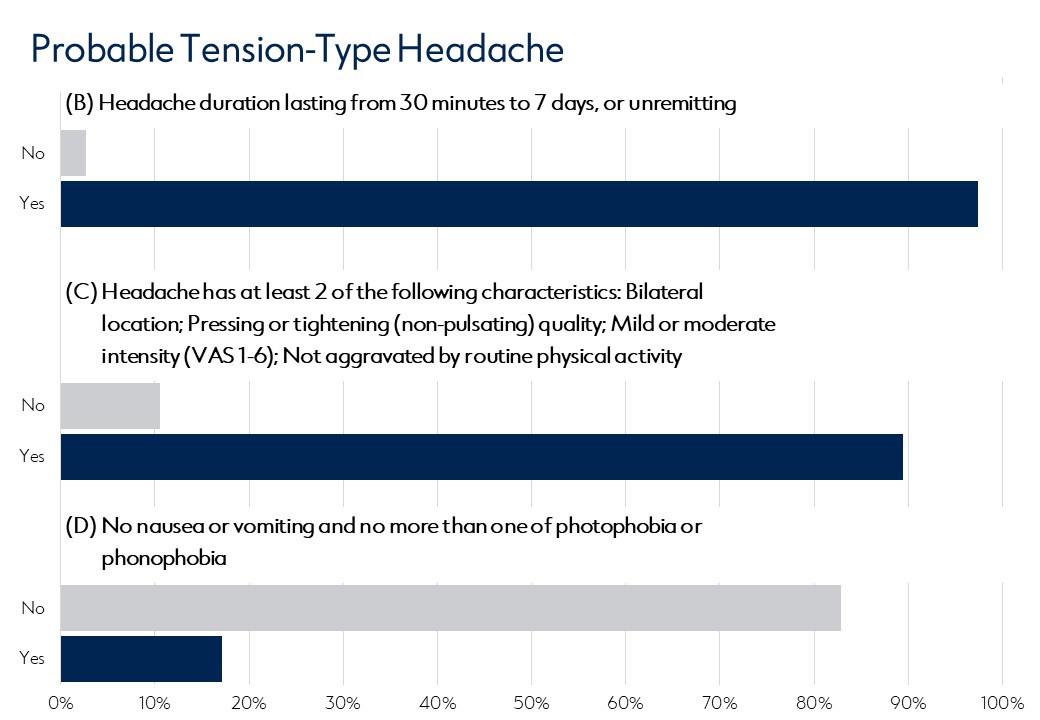


[B] Probable Tension-Type Headache


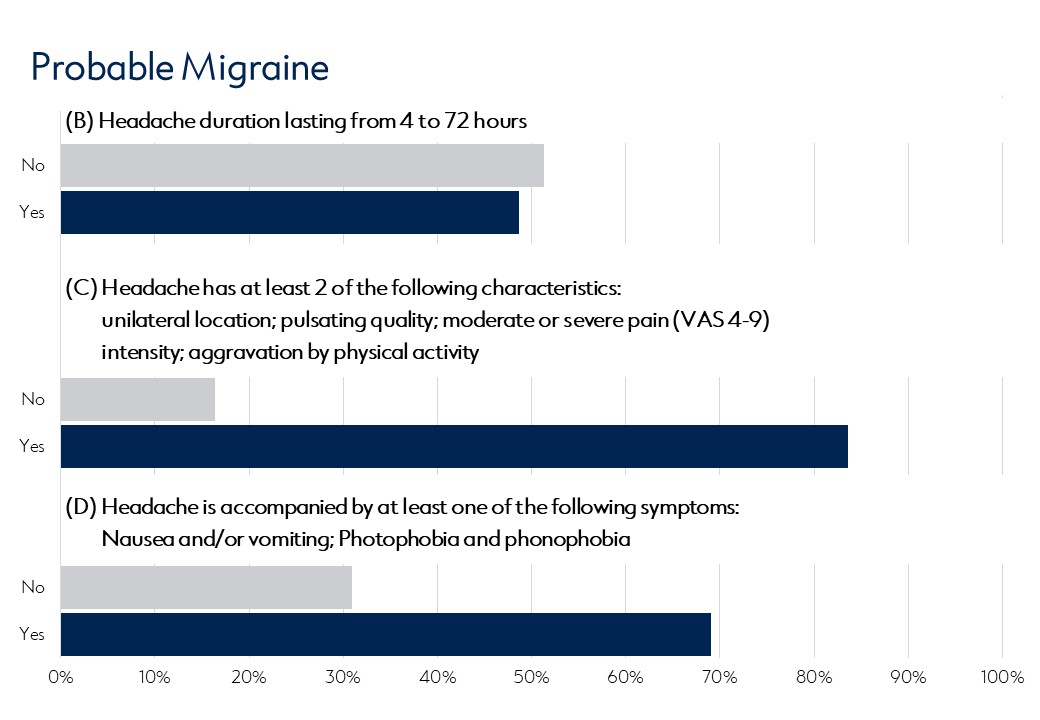


[A] Probable Migraine


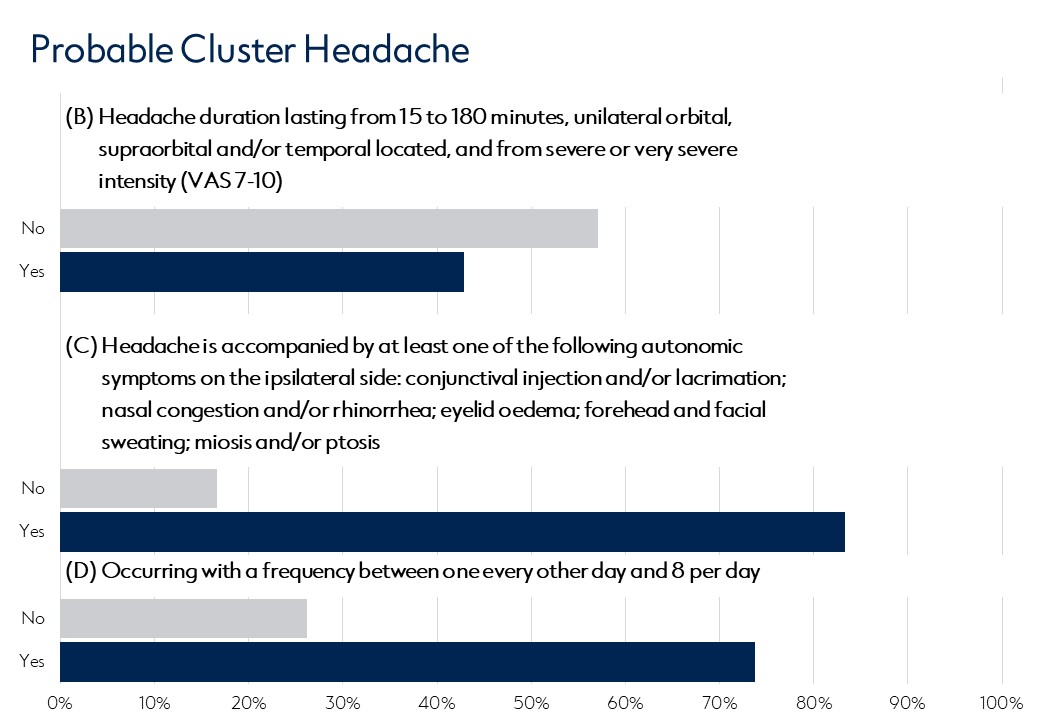


[C] Probable Cluster Headache

# sTable 1: Headache characteristics

| **Headache characteristics** | | |
| --- | --- | --- |
| Minimal attack duration | | 46.1 ± 113.1 |
| Maximal attack duration | | 75.3 ± 148.4 |
| Monthly headache days | | 14.7 ± 9.2 |
| Daily headache attacks | | 1.3 ± 5.7 |
| Daily headache attacks if more than one | | 12.5 ± 36 |
| Location | Unilateral | 1034 (64.2) |
|  | Bilateral | 540 (33.5) |
|  | Both Uni- and Bilateral | 37 (2.3) |
|  | | |
| **Aura** | | |
| Visually | | 383 (56.4) |
| Sensitive | | 77 (11.3) |
| Motor | | 14 (2.1) |
| Aphasic | | 60 (8.8) |
| Not Specified | | 6 (0.9) |
|  | | |
| **Pain aggravation by physical activity** | | |
| No | | 271 (20.2) |
| Yes | | 959 (71.4) |
| Sometimes | | 114 (8.5) |
| Pain intensity (Visual Analog Scale) | | 7 ± 1.8 |
|  | | |
| **Pain quality** | | |
| Pulsing | | 695 (43.7) |
| Dull | | 464 (29.1) |
| Cramping | | 4 (0.3) |
| Pounding | | 187 (11.7) |
| Pressing | | 828 (52) |
| Pulling | | 159 (10) |
| Stinging | | 532 (33.4) |
| Throbbing | | 98 (6.2) |
| Thumping | | 2 (0.1) |
|  | | |
| **Accompanying symptoms** | | |
| Photophobia | | 1115 (82.2) |
| Phonophobia | | 1077 (79.4) |
| Osmophobia | | 319 (23.5) |
| Nausea | | 1032 (76.1) |
| Vomiting | | 450 (33.2) |
| Dizziness | | 201 (14.8) |
|  | | |
| **Autonomic Symptoms** | | |
| No | | 746 (75.1) |
| Yes | | 236 (23.8) |
| Sometimes | | 11 (1.1) |
| Side | Contralateral | 10 (4.2) |
|  | Ipsilateral | 158 (66.9) |
|  | Both Sides | 6 (2.5) |
|  | Not Specified | 51 (26.3) |
| Watery Eye | | 158 (17) |
| Reddened Eye | | 87 (9.4) |
| Drooping Eyelid | | 57 (6.1) |
| Stuffy Nose | | 93 (10) |
| Runny Nose | | 91 (9.8) |
| Sweating | | 21 (2.3) |

####

#### sTable 2: Pre-existing headache diagnosis from the primary and secondary level of care

| **Diagnosis** | **Total cohort**  **N=1468** | **Primary**  **level of care**  **n=854** | **Secondary**  **level of care**  **n=614** | **p-value** |
| --- | --- | --- | --- | --- |
| Pre-existing diagnosis | 1020 (69.5) | 581 (68.0) | 439 (71.5) | 0.168 |
| None | 448 (30.5) | 273 (32.0) | 175 (28.5) | 0.437 |
| One diagnosis | 725 (49.4) | 408 (47.8) | 317 (51.6) |  |
| Two diagnoses | 268 (18.3) | 156 (18.3) | 112 (18.2) |  |
| Three diagnoses | 27 (1.8) | 17 (2.0) | 10 (1.6) |  |
|  |  |  |  |  |
| Migraine | 824 (56.1) | 458 (53.6) | 366 (59.6) | 0.025* |
| Tension-type headache | 365 (24.9) | 219 (25.6) | 146 (23.8) | 0.427 |
| Cluster headache | 111 (7.6) | 70 (8.2) | 41 (6.7) | 0.317 |
| Other headache | 42 (2.9) | 24 (2.8) | 18 (2.9) | 0.999 |
| Values are given in n (%)  * Statistically significant (p<0.05). | | | | |

####

#### sTable 3: Agreement of headache diagnosis from primary/secondary level of care relative to the tertiary level of care

| **Diagnosis from**  **Tertiary level of care** | **Pre-existing diagnosis from**  **primary/secondary care** | | | | | | **Agreement Statistics** | | |  |
| --- | --- | --- | --- | --- | --- | --- | --- | --- | --- | --- |
|  | **Agreement** | | | **Disagreement** | | | **Cohen's Kappa**  **(95% CI)** | **coefficient of determination (R^2^)** | **p-value** |  |
|  | **Positive agreement** | **Negative**  **agreement** | **Total**  **agreement** | **Positive disagreement** | **Negative disagreement** | **Total**  **disagreement** |  |  |  |  |
| Migraine | 800  54.5% | 276  18.8% | 1076  73.3% | 368  25.1% | 24  1.6% | 392  26.7% | 0.424  (0.375 - 0.473) | 0.180  (0.141 - 0.224) | <0.001* |  |
| TTH | 97  6.6% | 973  66.3% | 1070  72.9% | 130  8.9% | 268  18.3% | 398  27.1% | 0.169  (0.100 - 0.239) | 0.029  (0.010 - 0.057) | <0.001* |  |
| Cluster headache | 51  3.5% | 1346  91.7% | 1397  95.2% | 11  0.7% | 60  4.1% | 71  4.8% | 0.566  (0.468 - 0.665) | 0.320  (0.219 - 0.442) | <0.001* |  |
| **All diagnoses** | **948**  **21.5%** | **2595**  **58.9%** | **3543**  **80.4%** | **509**  **11.6%** | **352**  **8.0%** | **861**  **19.6%** | **0.546**  **(0.519 - 0.573)** | **0.298**  **(0.269 - 0.329)** | **<0.001*** |  |
| TTH = Tension-type headache, 95%CI = 95% confidence interval.  * Statistically significant (p<0.05). | | | | | | | | | | |

#### sTable 4: Agreement of headache diagnosis from primary/secondary level of care relative to the ICHD-3 Guidelines

| **ICHD-3 Diagnosis** | **All** | **All-but-one** | **Pre-existing diagnosis from**  **primary/secondary care** | | | | | | **Agreement Statistics** | | |
| --- | --- | --- | --- | --- | --- | --- | --- | --- | --- | --- | --- |
|  |  |  | **Agreement** | | | **Disagreement** | | | **Cohen's Kappa**  **(95% CI)** | **coefficient of determination (R^2^)** | **p-value** |
|  |  |  | **Positive agreement** | **Negative**  **agreement** | **Total**  **agreement** | **Positive disagreement** | **Negative disagreement** | **Total**  **disagreement** |  |  |  |
| Migraine | 1017 | 1237 | 806  54.9% | 328  22.3% | 1134  77.2% | 316  21.5% | 18  1.2% | 334  22.8% | 0.513 ^A^  (0.468 - 0.559) | 0.264  (0.219 - 0.313) | <0.001* |
| TTH | 59 | 363 | 144  9.8% | 1063  72.4% | 1207  82.2% | 40  2.7% | 221  15.1% | 261  17.8% | 0.430 ^A^  (0.367 - 0.492) | 0.184  (0.135 - 0.242) | <0.001* |
| Cluster headache | 36 | 78 | 54  3.7% | 1350  92.0% | 1404  95.6% | 7  0.5% | 57  3.9% | 64  4.4% | 0.607 ^B^  (0.513 - 0.701) | 0.368  (0.263 - 0.491) | <0.001* |
| **All diagnoses** | **1112** | **1678** | **1004**  **22.8%** | **2741**  **62.2%** | **3745**  **85.0%** | **363**  **8.2%** | **296**  **6.7%** | **659**  **15.0%** | **0.646 ^B^**  **(0.621 - 0.671)** | **0.417**  **(0.385 - 0.450)** | **<0.001*** |
| TTH = Tension-type headache, 95%CI = 95% confidence interval.  * Statistically significant (p<0.05). | | | | | | | | | | | |

#### sTable 5a: Agreement of headache diagnosis from primary level of care relative to the ICHD-3 Guidelines

| **ICHD-3 Diagnosis** | **All** | **All-but-one** | **Pre-existing diagnosis from**  **Primary level of care** | | | | | | **Agreement Statistics** | | |
| --- | --- | --- | --- | --- | --- | --- | --- | --- | --- | --- | --- |
|  |  |  | **Agreement** | | | **Disagreement** | | | **Cohen's Kappa**  **(95% CI)** | **coefficient of determination (R^2^)** | **p-value** |
|  |  |  | **Positive agreement** | **Negative**  **agreement** | **Total**  **agreement** | **Positive disagreement** | **Negative disagreement** | **Total**  **disagreement** |  |  |  |
| Migraine | 583 | 717 | 449  52.6% | 203  23.8% | 652  76.3% | 193  22.6% | 9  1.1% | 202  23.7% | 0.518  (0.445 - 0.591) | 0.268  (0.198 - 0.349) | <0.001* |
| TTH | 38 | 225 | 91  10.7% | 612  71.7% | 703  82.3% | 23  2.7% | 128  15.0% | 151  17.7% | 0.398  (0.296 - 0.500) | 0.158  (0.088 - 0.250) | <0.001* |
| Cluster headache | 22 | 42 | 31  3.6% | 780  91.3% | 811  95.0% | 4  0.5% | 39  4.6% | 43  5.0% | 0.669  (0.530 - 0.808) | 0.448  (0.281 - 0.653) | <0.001* |
| **All diagnoses** | **643** | **984** | **571**  **22.3%** | **1595**  **62.3%** | **2166**  **84.5%** | **220**  **8.6%** | **176**  **6.9%** | **396**  **15.5%** | **0.664**  **(0.627 - 0.702)** | **0.441**  **(0.393 - 0.492)** | **<0.001*** |
| TTH = Tension-type headache, 95%CI = 95% confidence interval.  * Statistically significant (p<0.05). | | | | | | | | | | | |

#### sTable 5b: Agreement of headache diagnosis from secondary level of care relative to the ICHD-3 Guidelines

| **ICHD-3 Diagnosis** | **All** | **All-but-one** | **Pre-existing diagnosis from**  **Secondary level of care** | | | | | | **Agreement Statistics** | | |
| --- | --- | --- | --- | --- | --- | --- | --- | --- | --- | --- | --- |
|  |  |  | **Agreement** | | | **Disagreement** | | | **Cohen's Kappa**  **(95% CI)** | **coefficient of determination (R^2^)** | **p-value** |
|  |  |  | **Positive agreement** | **Negative**  **agreement** | **Total**  **agreement** | **Positive disagreement** | **Negative disagreement** | **Total**  **disagreement** |  |  |  |
| Migraine | 434 | 520 | 357  58.1% | 125  20.4% | 482  78.5% | 123  20.0% | 9  1.5% | 132  21.5% | 0.509  (0.450 - 0.568) | 0.259  (0.202 - 0.323) | <0.001* |
| TTH | 21 | 138 | 53  8.6% | 451  73.5% | 504  82.1% | 17  2.8% | 93  15.1% | 110  17.9% | 0.450  (0.370 - 0.530) | 0.202  (0.137 - 0.280) | <0.001* |
| Cluster headache | 14 | 36 | 23  3.7% | 570  92.8% | 593  96.6% | 3  0.5% | 18  2.9% | 21  3.4% | 0.567  (0.441 - 0.693) | 0.321  (0.194 - 0.480) | <0.001* |
| **All diagnoses** | **469** | **694** | **433**  **23.5%** | **1146**  **62.2%** | **1579**  **85.7%** | **143**  **7.8%** | **120**  **6.5%** | **263**  **14.3%** | **0.632**  **(0.599 - 0.666)** | **0.400**  **(0.359 - 0.443)** | **<0.001*** |
| TTH = Tension-type headache, 95%CI = 95% confidence interval.  * Statistically significant (p<0.05). | | | | | | | | | | | |

#### sTable 6: Agreement of diagnosis from headache specialist according to the ICHD-3 Guidelines

| **ICHD-3 Diagnosis** | **All** | **All-but-one** | **Diagnosis from**  **Tertiary level of care** | | | | | | **Agreement Statistics** | | |
| --- | --- | --- | --- | --- | --- | --- | --- | --- | --- | --- | --- |
|  |  |  | **Agreement** | | | **Disagreement** | | | **Cohen's Kappa**  **(95% CI)** | **coefficient of determination (R^2^)** | **p-value** |
|  |  |  | **Positive agreement** | **Negative**  **agreement** | **Total**  **agreement** | **Positive disagreement** | **Negative disagreement** | **Total**  **disagreement** |  |  |  |
| Migraine | 1017 | 1237 | 1163  79.2% | 296  20.2% | 1459  99.4% | 4  0.3% | 5  0.3% | 9  0.6% | 0.981  (0.969 - 0.993) | 0.963  (0.939 - 0.987) | <0.001* |
| TTH | 59 | 363 | 214  14.6% | 1237  84.3% | 1451  98.8% | 4  0.3% | 13  0.9% | 17  1.2% | 0.955  (0.934 - 0.976) | 0.912  (0.872 - 0.953) | <0.001* |
| Cluster headache | 36 | 78 | 61  4.2% | 1406  95.8% | 1467  99.9% | 0  0.0% | 1  0.1% | 1  0.1% | 0.992  (0.975 – 0.999) | 0.983  (0.95 - 0.998) | <0.001* |
| **All diagnoses** | **1112** | **1678** | **1438**  **32.7%** | **2939**  **66.7%** | **4377**  **99.4%** | **8**  **0.2%** | **19**  **0.4%** | **27**  **0.6%** | **0.986**  **(0.981 - 0.991)** | **0.972**  **(0.962 - 0.983)** | **<0.001*** |
| TTH = Tension-type headache, 95%CI = 95% confidence interval.  * Statistically significant (p<0.05). | | | | | | | | | | | |

#### sTable 7: Predictors for a positive disagreement in diagnosing migraine in the PSLC according to the ICHD-3 criteria, n=1042

| **Clinical migraine features as mentioned in the ICHD-3** | | **Positive disagreement**  **(missed diagnosis)** | | **Univariate** | **Multivariate** |  |
| --- | --- | --- | --- | --- | --- | --- |
|  |  | **No**  **N=708** | **Yes n=334** | **OR (95% CI)**  **p-value** | **OR (95% CI)**  **p-value** |  |
| Attack duration 4 - 72 hours | No | 42 (66.7) | 21 (33.3) | 1.06 (0.62 - 1.83)  0.822 |  |  |
|  | Yes | 666 (68) | 313 (32) | rev. |  |  |
| Pain location unilateral | No | 82 (73.9) | 29 (26.1) | 0.73 (0.47 - 1.13)  0.158 |  |  |
|  | Yes | 626 (67.2) | 305 (32.8) | rev. |  |  |
| Pain quality pulsating | No | 107 (66.9) | 53 (33.1) | 1.06 (0.74 - 1.52)  0.752 |  |  |
|  | Yes | 601 (68.1) | 281 (31.9) | rev. |  |  |
| Pain intensity VAS 4-9 | No | 116 (51.3) | 110 (48.7) | 2.51 (1.85 - 3.39)  <0.001* | 2.27 (1.67 - 3.1)  <0.001* |  |
|  | Yes | 592 (72.5) | 224 (27.5) | rev. | rev. |  |
| Pain aggravation by PA | No | 298 (63.8) | 169 (36.2) | 1.41 (1.09 - 1.83)  0.010* |  |  |
|  | Yes | 410 (71.3) | 165 (28.7) | rev. |  |  |
| Photophobia | No | 71 (57.3) | 53 (42.7) | 1.69 (1.15 - 2.48)  0.007* | 1.58 (1.06 - 2.34)  0.024* |  |
|  | Yes | 637 (69.4) | 281 (30.6) | rev. | rev. |  |
| Phonophobia | No | 90 (61.6) | 56 (38.4) | 1.38 (0.96 - 1.99)  0.079 |  |  |
|  | Yes | 618 (69) | 278 (31) | rev. |  |  |
| Nausea | No | 91 (55.8) | 72 (44.2) | 1.86 (1.32 - 2.62)  <0.001* | 1.5 (1.04 - 2.17)  0.030* |  |
|  | Yes | 617 (70.2) | 262 (29.8) | rev. | rev. |  |
| Vomiting | No | 404 (63.4) | 233 (36.6) | 1.74 (1.32 - 2.29)  <0.001* | 1.54 (1.14 - 2.07)  0.004* |  |
|  | Yes | 304 (75.1) | 101 (24.9) | rev. | rev. |  |
| Aura | No | 395 (63.5) | 227 (36.5) | 1.68 (1.28 - 2.21)  <0.001* | 1.7 (1.28 - 2.26)  <0.001* |  |
|  | Yes | 313 (74.5) | 107 (25.5) | rev. | rev. |  |
| OR = Odds Ratio; 95% CI = 95% Confidence interval.  * Statistically significant (p<0.05). | | | | | | |

#### sTable 8: Predictors for a negative diagnostic disagreement of TTH in the PSLC according to the ICHD-3 criteria, n=1544

| **Clinical TTH features as mentioned in the ICHD-3** | | **Negative disagreement** | | **Univariate** | **Multivariate** | |
| --- | --- | --- | --- | --- | --- | --- |
|  |  | **No**  **n=1362** | **Yes n=182** | **OR (95% CI)**  **p-value** | **OR (95% CI)**  **p-value** | |
| Duration 30 min - 7 days or   unremitting | No | 63 (87.5) | 9 (12.5) | 1.07 (0.52 - 2.2)  0.848 |  | |
|  | Yes | 1299 (88.2) | 173 (11.8) | rev. |  | |
| Pain location bilateral | No | 892 (85.5) | 151 (14.5) | 2.57 (1.72 - 3.84)  <0.001* | 1.69 (1.11 - 2.57)  0.014* | |
|  | Yes | 470 (93.8) | 31 (6.2) | rev. | rev. | |
| Pain quality pressing | No | 896 (85.3) | 154 (14.7) | 2.86 (1.88 - 4.34)  <0.001* | 2.12 (1.38 - 3.26)  0.001* | |
| or tightening (non-pulsating) | Yes | 466 (94.3) | 28 (5.7) | rev. | rev. | |
| Pain intensity VAS 1 -6 | No | 920 (85.6) | 155 (14.4) | 2.76 (1.8 - 4.22)  <0.001* | 2.02 (1.31 - 3.12)  0.001* | |
|  | Yes | 442 (94.2) | 27 (5.8) | rev. | rev. | |
| No pain aggravation by PA | No | 1148 (86.7) | 176 (13.3) | 5.47 (2.39 - 12.5)  <0.001* | 3.6 (1.56 - 8.33)  0.003* | |
|  | Yes | 214 (97.3) | 6 (2.7) | rev. | rev. | |
| Photophobia | No | 431 (92.7) | 34 (7.3) | 0.5 (0.34 - 0.73)  <0.001* |  | |
|  | Yes | 931 (86.3) | 148 (13.7) | rev. |  | |
| Phonophobia | No | 446 (92) | 39 (8) | 0.56 (0.39 - 0.81)  0.002* |  | |
|  | Yes | 916 (86.5) | 143 (13.5) | rev. |  | |
| Nausea | No | 473 (92.4) | 39 (7.6) | 0.51 (0.35 - 0.74)  <0.001* |  | |
|  | Yes | 889 (86.1) | 143 (13.9) | rev. |  | |
| Vomiting | No | 976 (89.2) | 118 (10.8) | 0.73 (0.53 - 1.01)  0.058 |  | |
|  | Yes | 386 (85.8) | 64 (14.2) | rev. |  | |
| PA = physical activity; OR = Odds Ratio; 95% CI = 95% Confidence interval.  * Statistically significant (p<0.05). | | | | | |  |

#### sTable 9: Predictors for a negative diagnostic disagreement of CH in the PSLC according to the ICHD-3 criteria, n=1600

| **Clinical migraine features as mentioned in the ICHD-3** | | **Negative disagreement** | | **Univariate** | **Multivariate** | |
| --- | --- | --- | --- | --- | --- | --- |
|  |  | **No**  **N=1536** | **Yes n=64** | **OR (95% CI)**  **p-value** | **OR (95% CI)**  **p-value** | |
| Attack duration 15 - 180 minutes | No | 1480 (95.9) | 63 (4.1) | 2.38 (0.32 - 17.5)  0.393 |  | |
|  | Yes | 56 (98.2) | 1 (1.8) | rev. |  | |
| Pain location unilateral | No | 552 (97.7) | 13 (2.3) | 0.45 (0.24 - 0.84)  0.012* | 0.46 (0.24 - 0.87)  0.017* | |
|  | Yes | 984 (95.1) | 51 (4.9) | rev. | rev. | |
| Pain Intensity VAS 7 - 10 | No | 593 (96.9) | 19 (3.1) | 0.67 (0.39 - 1.16)  0.153 |  | |
|  | Yes | 943 (95.4) | 45 (4.6) | rev. |  | |
| Presence of Ipsilateral | No | 1417 (96.4) | 53 (3.6) | 0.4 (0.21 - 0.8)  0.009* | 0.49 (0.25 - 0.98)  0.043* | |
| autonomic symptoms | Yes | 119 (91.5) | 11 (8.5) | rev. | rev. | |
| Attack frequency of one every | No | 931 (96.5) | 34 (3.5) | 0.74 (0.45 - 1.22)  0.232 | 0.65 (0.39 - 1.08)  0.094 | |
| other day - 8 per day | Yes | 605 (95.3) | 30 (4.7) | rev. | rev. | |
| OR = Odds Ratio; 95% CI = 95% Confidence interval; VAS = Visual Analog Scale.  * Statistically significant (p<0.05). | | | | | |  |
